# Prediction and Experimental Validation of a New Salinity-Responsive Cis-Regulatory Element (CRE) in a Tilapia Cell Line

**DOI:** 10.3390/life12060787

**Published:** 2022-05-25

**Authors:** Chanhee Kim, Xiaodan Wang, Dietmar Kültz

**Affiliations:** 1 Stress-Induced Evolution Laboratory, Department of Animal Sciences, University of California, Davis, CA 95616, USA; cshkim@ucdavis.edu; 2 Laboratory of Aquaculture Nutrition and Environmental Health, School of Life Sciences, East China Normal University, Shanghai 200241, China; xdwang@bio.ecnu.edu.cn

**Keywords:** cellular osmoregulation, cis-regulatory element, salinity, stress tolerance, fish, transcription factor, transcriptional regulation

## Abstract

Transcriptional regulation is a major mechanism by which organisms integrate gene x environment interactions. It can be achieved by coordinated interplay between cis-regulatory elements (CREs) and transcription factors (TFs). Euryhaline tilapia (*Oreochromis mossambicus*) tolerate a wide range of salinity and thus are an appropriate model to examine transcriptional regulatory mechanisms during salinity stress in fish. Quantitative proteomics in combination with the transcription inhibitor actinomycin D revealed 19 proteins that are transcriptionally upregulated by hyperosmolality in tilapia brain (OmB) cells. We searched the extended proximal promoter up to intron1 of each corresponding gene for common motifs using motif discovery tools. The top-ranked motif identified (STREME1) represents a binding site for the Forkhead box TF L1 (FoxL1). STREME1 function during hyperosmolality was experimentally validated by choosing two of the 19 genes, chloride intracellular channel 2 (*clic2*) and uridine phosphorylase 1 (*upp1*), that are enriched in STREME1 in their extended promoters. Transcriptional induction of these genes during hyperosmolality requires STREME1, as evidenced by motif mutagenesis. We conclude that STREME1 represents a new functional CRE that contributes to gene x environment interactions during salinity stress in tilapia. Moreover, our results indicate that FoxL1 family TFs are contribute to hyperosmotic induction of genes in euryhaline fish.

## 1. Introduction

A major challenge of biology is understanding the mechanisms that govern gene x environment interactions and the phenotypic diversity of organisms. Studies of physiological and biochemical ecology aimed at understanding and explaining how organisms adapt to environmental change and stress currently rely heavily on correlations of phenotypes with particular single nucleotide polymorphisms (SNPs) or other sequence variation and transcriptomics [1]. However, multiple levels of biological organization and regulation are interspersed between the genome and complex phenotypes with transcriptional regulation of gene expression being only one of many mechanisms by which changes in transcriptomes, proteomes, and complex cellular and organismal phenotypes are achieved [2]. 

One mechanism by which organisms respond to environmental signals (e.g., temperature changes, salinity fluctuations, etc.) is by regulating gene expression [3,4]. Transcriptional regulation of specific gene(s) is a fundamental regulatory process for controlling gene expression [5]. Understanding transcriptional regulation is thus critical for elucidating how molecular mechanisms shape the phenotypic changes of organisms in response to environmental stress [6]. Transcriptional regulation is based on the interaction of cis-regulatory elements (CREs) that control the transcription of associated genes and transcription factors (TFs) that recognize and bind to CREs to influence transcription of those genes [7]. Harmonious interactions (binding events) between those two components in response to environmental stimuli are known to govern gene expression in an organized manner [8,9,10]. Despite much attention and interest in environmental control of gene expression and many studies documenting elaborate changes of transcriptomes in response to environmental stresses, little is known about the molecular mechanisms that control transcriptional regulation in stress tolerant (eurytopic) organisms exposed to environmental stress.

Mozambique tilapia (*Oreochromis mossambicus*) are eurytopic fish that are highly tolerant to many environmental stresses, including large salinity changes. Gene expression patterns of tilapia have been correlated with various phenotypic characteristics that are important for aquaculture, e.g., muscle growth and meat quality [11,12]. Another important trait for tilapia aquaculture is environmental resilience. Several tilapia species, including *O. mossambicus*, have undergone a remarkable adaptive evolution to cope with large salinity fluctuations in their environment. *O. mossambicus* is able to tolerate salinities from 0 to 120 g/kg and plasma osmolality changes ranging from 305 to 800 mOsmol/kg [13]. This astonishing phenotypic plasticity renders Mozambique tilapia an excellent model for investigating the underlying molecular mechanisms that orchestrate the control of gene expression during hyperosmotic salinity stress. 

The influence of salinity on gene expression patterns in tilapia has been investigated, complemented by studies of other systems-level, holistic molecular phenotypes, notably metabolomes and proteomes [14,15,16]. These systems-level studies have revealed that salinity stress has very pronounced effects on transcriptomes and proteomes, causing significant changes in hundreds of gene products. Although these studies have correlated many gene products with salinity stress in tilapia and other euryhaline fish, the regulatory mechanisms that are causal for such changes are mostly elusive. Few studies have identified the mechanism of regulation of transcripts and proteins, i.e., whether regulation takes place at the level of transcription (gene expression), posttranscriptional mRNA abundance regulation, translational regulation, or protein turnover. We have previously demonstrated that gene expression control by a specific novel CRE, the osmotic/salinity response element 1 (OSRE1), is largely responsible for the hyperosmotic upregulation of several osmoregulated proteins [17,18]. 

Approaches for identifying and experimentally validating regulatory sequences, such as CREs of a particular gene that mediate a response to environmental stress (e.g., temperature and salinity), have been mostly used for relatively few model species [19,20]. They require robust genomic resources and are laborious and technically challenging. However, as more genomic sequence information and effective computational tools have become available for a greater diversity and number of species, genome-wide comparative approaches for identifying potential regulatory sequences such as CREs have become more powerful and are now commonly used for yeast, certain plants, and mammalian models [10,21,22,23]. The combination of computational prediction and experimental validation represents a powerful tool for elucidating the mechanisms that underlie changes in gene expression in response to environmental or developmental cues and for establishing causality between changes in certain transcript and protein abundances and environmentally controlled signaling networks [24,25]. Recently, such approaches have been used to delineate transcriptional networks in zebrafish and understand genetic networks that control the physiological adaptation of fish [26,27]. In one study, differentially expressed genes of zebrafish (*Danio rerio*) exposed to low temperature were analyzed for enriched CREs using a motif discovery program, and subsequent experimental validation of the identified motifs revealed cis- and trans-elements (CREs and TFs) that control gene expression during cold stress [26]. In another study, tilapia (*Oreochromis niloticus*) and zebrafish (*D. rerio*) were compared to decipher divergent aspects of cold stress responses by identifying TF binding sites in extended promoter region of genes with species-specific regulation during cold stress. This approach was complemented by experimental validation and yielded a genetic network of cold stress responses in different fish species [27]. 

In this study, a similar comparative bioinformatics approach was used to identify a novel CRE and corresponding TF candidate, and then experimentally validate the functionality of the candidate CRE during hyperosmotic stress.

## 2. Materials and Methods

### 2.1. Hyperosmotic Stress Challenge and Actinomycin D Treatment

The *O. mossambicus* brain (OmB) cell line was subjected to all hyperosmotic stress challenges. L-15 medium containing 5% (vol/vol) fetal bovine serum (FBS) and 1% (vol/vol) penicillin-streptomycin at 26 °C was used to grow OmB cells at 2% CO2 as previously described [17,18]. Using a large supply of OmB cell superstock (passage 15; P15), all experiments were conducted on OmB cells between P20 and P27. OmB cells were passaged every 3–4 days using a 1:5 splitting ratio and exposed to hyperosmotic medium (osmolality: 650 mOsmol/kg) during hyperosmotic stress challenge. The hyperosmotic medium was made by adding an appropriate volume of hyperosmotic stock solution (osmolality: 2820 mOsmol/kg) to isosmotic L15 medium (osmolality: of 315 mOsmol/kg). An appropriate amount of NaCl was added to isosmotic L-15 medium to prepare the hyperosmotic stock solution. Medium osmolality was measured by freezing point micro-osmometer (Advanced Instruments). All exposures were performed by acutely increasing medium osmolality from 315 to 650 mOsmol/kg for 24 h. Parallel handling controls were subjected to medium change without increasing the medium osmolality. Actinomycin D, a widely-used transcription initiation inhibitor [28,29], was added at a concentration of 10 µM to a subset of hyperosmotically challenged OmB cells and isosmotic controls to analyze the contribution of transcriptional regulation in the hyperosmotic upregulation of protein. 

### 2.2. Quantitative Proteomics 

Sample preparation and in-solution trypsin digestion were performed as previously described [30]. A DIA-LCMS2 approach was used to ensure highly accurate relative quantitation of many proteins. DIA was invented in 2012 [31,32] and avoids undersampling of peaks and inconsistent peak picking. DIA-LCMS2 is also known under the acronym sequentially windowed acquisition of all theoretically possible MS2 spectra (SWATH)-MS [33,34] and represents a merger of targeted MS approaches such as selected reaction monitoring (SRM) and non-targeted MS2 spectra acquisition [35]. DIA targeting of specific transitions, precursors, peptides, and proteins is performed post-acquisition by interrogating all MS2 spectra present in a sample against a previously validated DIA assay library. Using a previously published procedure [30], we have generated a high quality DIA assay (MS2 spectral) library for *O. mossambicus* OmB cells which includes 3043 unique proteins meeting stringent quality control (QC) criteria and consisting of non-redundant diagnostic peptides (Figure S1). DIA data were acquired as previously described [36] and analyzed with Skyline [37], mProphet [38], MSstats [39]. They were deposited and are publicly accessible at PanoramaPublic [40] and ProteomeXchange [41] (see Data Availability Statement). The following parameters were used for MSstats analysis of quantitative DIA data: normalization method = equalize medians, confidence interval = 95%, scope = protein, summary method = Tukey’s median polish, mProphet Q value cutoff = 0.05. 

### 2.3. Motif Discovery and Refinement 

Motif-based sequence analyses were performed using the MEME bioinformatics suite [42]. Specifically, three MEME suite analysis tools were used: STREME [43], TOMTOM [44], and FIMO [45]. Common motifs in a set of the regulatory sequences were searched for using STREME, a motif discovery tool that identifies motifs, which are enriched in the input sequences (regulatory sequences from 19 transcriptionally osmoregulated tilapia genes). STREME compares the input sequences to a control dataset that is generated by shuffling each of the input sequences. Approximately 5 kb representing the extended promoters up to intron 1 were extracted in FASTA format for each of the 19 genes using the genome database of *O. niloticus* (isolate F11D_XX linkage groupS, O_niloticus_UMD_NMBU, whole genome shotgun sequence, NCBI). STREME was carried out on these 19 regulatory sequences using default parameters, except the range was set to between 8 and 18 bp to capture pertinent potential transcription factor binding sites according to the typical length range of binding sites for TFs [46,47]. To estimate false discovery rate, STREME processes both the input sequences and an equally large decoy set consisting of their reverse complements. This approach permits the use of Fisher’s exact test as a statistical test for assessing statistical significance of motif enrichment. Significant STREME motifs identified with this approach were then evaluated with the TOMTOM motif comparison tool to compare these motifs with known TF binding sites (CREs). In the TOMTOM approach, to sequences were aligned to curated eukaryotic DNA-JASPAR, vertebrate, and UniPROBE mouse databases of known CREs with a *p*-value cut-off of 5e-3 and sequence divergence cutoff of fewer than 2 bases. FIMO was subsequently run to scan and annotate all occurrences of TOMTOM-annotated motifs in each regulatory region of the 19 hyperosmotically induced genes. The FIMO tool converts each input motif into a log-odds position specific scoring matrix (PSSM) and uses each PSSM to independently scan each input sequence. All positions in each sequence that match a motif with a statistically significant log-odds score are then reported. The q-value is similar to a *p*-value but corrected for multiple testing, and a q-value of 0.01 or less was used as the threshold for statistical significance using FIMO. 

### 2.4. Cloning 

Genomic DNA used for PCR amplification was extracted from OmB cells using the PureLink Genomic DNA mini kit (Thermo Fisher Scientific, Waltham, MA, USA). PCR primers were designed using Geneious Prime 2022.0.1 (Biomatters, https://www.geneious.com) with the *O. niloticus* genomic sequences of chloride intracellular channel 2 (NCBI Gene ID # 100694858) and uridine phosphorylase 1 (NCBI Gene ID # 100690403) as templates. A CCCCC spacer followed by a restriction enzyme recognition site was added to the 5’ end of each PCR primer. The restriction enzymes KpnI and NcoI (New England Biolabs, Ipswich, MA, USA) were used to clone PCR amplicons representing extended proximal promoters up to intron 1 of each gene into pBS_EGFP expression vector. Q5 high-fidelity DNA polymerase (New England Biolabs, Ipswich, MA, USA) and GoTaq Green Master Mix (Promega, Madison, WI, USA) were used to amplify DNA fragments after confirming single-band PCR amplicons on regular DNA agarose gels. PCR was carried out as follows: initial denaturation at 95 °C for 3 min followed by 35 cycles of 95 °C for 30 s, annealing: 50–60° for 30 s, elongation: 72 °C for 0.5–1 min and 72 °C for 15 min. Annealing temperature and extension time were adjusted according to the chemical features of the primers (e.g., Tm) and the lengths of amplicons. PCR amplicons were checked by agarose gel electrophoresis and subsequently purified using the PureLink PCR Purification Kit (Thermo Fisher Scientific) if a single band was detected. In cases where multiple bands were visible on an agarose gel, a specific band with the expected size was gel-extracted using the QIAquick Gel Extraction Kit (Qiagen, Hilden, Germany). Specific primers were designed to clone the parts harboring predicted motifs within proximal regulatory sequences of each gene. Primer pairs were generated to be compatible with KpnI and NcoI sites in the acceptor expression (clic2_5’KpnI, clic2_3’NcoI, upp1_5’KpnI, upp1_3’NcoI, the corresponding primer sequences are listed in Table S1). 

PCR amplicons and pBS_EGFP vector were double digested with KpnI and NcoI restriction enzymes. Restriction enzyme digestion reactions were prepared by adding the following ingredients to PCR amplicons and pBS_EGFP vector serving as DNA templates: 10 μL reaction buffer (rCutSmartBuffer^TM^ and NEBuffer^TM^ r1.1), 2 μL (10 U/μL) of each restriction enzyme, 0.5–2 μg of purified PCR amplicons (or pBS_EGFP vector), and nuclease-free H_2_O were added to yield 100 μL final reaction volume. After overnight incubation at 37 °C to ensure complete digestion, reactions were stopped by heating at 80 °C for 20 min. Digested PCR amplicon inserts and vectors were purified using the PureLink Quick PCR Purification Kit (Thermo Fisher Scientific) and subsequently ligated to produce desired recombinant constructs for experimental validation using T4 DNA ligase (Thermo Fisher Scientific). Ligation reactions were prepared as follows: 50 ng of vector, 10–20 ng of insert (1:5 molar ratio), 2 μL of ligase buffer, 1 μL of T4 ligase (1 U/μL) and nuclease-free H_2_O were added to 20 μL of digested materials. Ligation reactions were incubated in a thermocycler (Mastercycler, Eppendorf) at 25 °C for 5 h. The ligation products were transformed into 10-beta-competent *E. coli* (New England Biolabs) as previously described [18]. After transformation, an appropriate amount of the bacterial solution was spread onto a prewarmed (37 °C) LB-ampicillin plate. The plate was used for single colony picking and subsequent colony PCR to check for the presence of intended PCR amplicon inserts. 

Colony PCR was performed by heating tubes containing a single bacterial clone picked from the plates at 95 °C for 15 min and quick centrifugation, and resulting supernatants were used as a template. The supernatant (3 μL) was mixed with forward (M13_Forward) and reverse primers (GFP_R5) that flank the corresponding PCR amplicon insert (Table S1). Colony PCR thermocycler conditions were the same as described above and amplicons were confirmed by agarose gel electrophoresis. Colonies that harbored an insert of the expected size were chosen for bacterial cell cultures followed by plasmid purification. Each bacterial colony was inoculated into liquid LB medium and grown for 18–20 h to obtain a sufficient amount of plasmid. Liquid cultures were harvested and purified according to manufacturer’s protocol using endotoxin-free PureLink Quick Plasmid Miniprep Kit (Thermo Fisher Scientific, Waltham, MA, USA). Insert sequences in purified DNA constructs were verified by Sanger sequencing with M13_Forward and GFP_R5 primers at the University of California, Davis DNA Sequencing Facility. 

### 2.5. Site-Directed Motif Mutagenesis 

“Overlap Extension PCR” was used to mutate candidate motifs identified in regulatory regions of the tilapia *clic2* and *upp1* genes. Two or three independent PCR amplifications were performed using the extended PCR primers containing nucleotide replacements for introducing nonfunctional motifs and complementary sequences for stable binding into sequence fragments. PCR amplicons representing fragments of the overall sequence were then then used as PCR templates (1 µL of each PCR amplicon) and subsequently stitched together using PCR with the outermost primers to obtain a single intermediate PCR amplicon. The final amplifications of the entire 1 kb long regulatory regions containing the mutated motifs of *clic2* and *upp1* were then performed using the same PCR primers as those used for amplification of the corresponding wildtype sequences (Table S1). The sequences for all mutagenesis constructs for *clic2* and *upp1* were confirmed by Sanger sequencing after each plasmid was miniprepped as described above (Figure S1). 

### 2.6. Quantitative Fluorescent Reporter Assay

To perform quantitative fluorescent reporter assays, tilapia OmB cells were seeded in six-well plates (Corning, Glendale, AZ, USA) and transiently transfected at 80% confluency with four different enhanced green fluorescent protein (GFP) expression vectors containing regulatory regions of *clic2* (wildtype and mutant) and *upp1* (wildtype and mutant). After 24 h, the transfected cells were dosed either with hyperosmotic (650 mOsmol/kg) or isosmotic (315 mOsmol/kg) medium for 24 h. For GFP quantification, a Dmi8 fluorescence microscope (Leica) was used to capture fluorescence micrographs of OmB cells cotransfected with one of the GFP-expression vectors and a control vector containing red fluorescent protein (RFP) that was used for normalization. Instead of capturing a random single fluorescence image of part of the each well, a complete tile scan of the well was performed to quantify fluorescence in the entire well for all conditions using the Dmi8 automatic stage microscope (Leica, Wetzlar, Germany) and Leica Application Suite X (LAS X) software. Tile scanning of each well was carried out to detect GFP and RFP signals from the designated part of each well. Intensity sum values were used to calculate the relative GFP/RFP intensity ratio. Five independent biological replicates (individual wells) were used to enable testing for statistical significance of treatment effects on GFP/RFP intensity ratio. 

## 3. Results

### 3.1. Transcriptional Regulation Is Required for Upregulation of Proteins in OmB Cells Exposed to Hyperosmotic Stress

**Figure 1:**
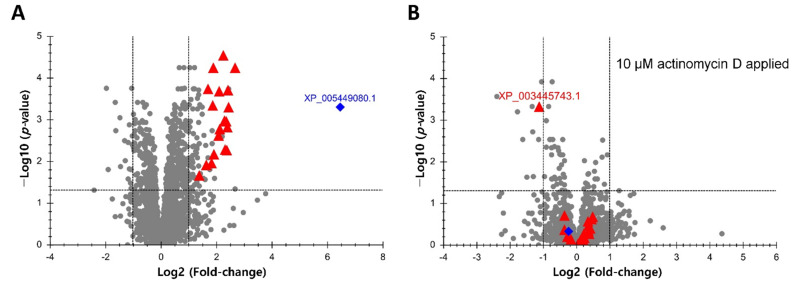
Relative abundances of 3043 *Oreochromis mossambicus* proteins in OmB cells exposed for 24 h to hyperosmotic stress (650 mOsmol/kg) versus isosmotic media (315 mOsmol/kg). (**A**) Volcano plot indicating the 19 significantly up-regulated proteins that were selected for comparative sequence analyses and motif searches (red triangles). The x axis displays the fold change of protein abundance in hyperosmotic versus isosmotic medium on a log 2 scale. The y axis displays the negative decadic logarithm of the MSstats-adjusted (multiple testing corrected) *p* value. Inositol monophosphatase (blue diamond) was not included in this set because its FC was much greater than that of the other proteins’ and it had been analyzed previously in depth [17]. (**B**) Volcano plot for the same proteins as shown in panel A except that OmB cells were exposed to hyperosmolality in the presence of 10 µM of the transcriptional inhibitor actinomycin D. Data are based on five replicates for each treatment and control group. For accession numbers of all 19 proteins indicated by red triangles and used for further analyses by motif searching please refer to Figure 2.

Increases in protein abundances of Mozambique tilapia OmB cells exposed for 24 h to hyperosmolality (650 mOsmol/kg) compared to isosmotic controls (315 mOsmol/kg) were calculated based on DIA data using Skyline and MSstats and visualized using volcano plots. Remarkably, the upregulation of all statistically significant proteins (multiple testing correct *p* < 0.05 and fold-change > 2) was abolished when transcription was inhibited by the inclusion of 10 µM actinomycin D in the medium (Figure 1). This result confirms transcriptional regulation as a predominant mechanism underlying the upregulation of proteins during hyperosmotic stress. Nineteen hyperosmotically upregulated proteins whose upregulation was completely abolished by transcription inhibition were chosen to serve as a basis for identifying common motifs in their regulatory sequences. These proteins are indicated as red triangles in Figure 1. Inositol monophosphatase (XP_005449080.1) was not included in this set even though it showed the same pattern of regulation (blue diamond in Figure 1) because the extent of upregulation was more than an order of magnitude greater than for the other proteins and we had previously analyzed the regulation of this protein and its corresponding gene in depth [17]. Interestingly, one of the 19 proteins selected for motif searching (ferritin, heavy subunit, XP_003445743.1) was significantly downregulated in the presence of actinomycin D, suggesting that it may be subject to very rapid turnover in OmB cells exposed to hyperosmolality.

### 3.2. Discovery of Putative CRE Motifs That Mediate Hyperosmotic Induction of Tilapia Genes 

**Figure 2:**
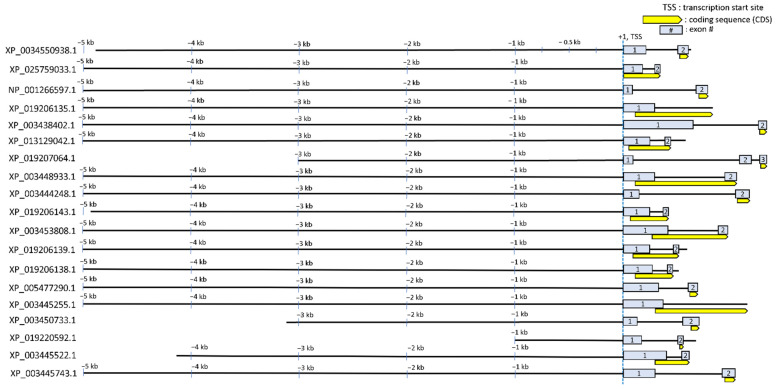
Schematic genomic landscape of the regulatory sequences of 19 hyperosmotically induced tilapia genes. Each of the regulatory sequences up to 5 kb upstream (some genes have upstream regulatory regions less than 5 kb due to overlapping with gene body of other genes) and 5’ UTR (up to CDS) are depicted as black lines. Light grey boxes indicate exons and the yellow boxes with one-sided arrow indicate the coding sequence (CDS) of each gene including intron 1 if applicable. Each sequence is labeled with the NCBI accession number of the corresponding protein. Relative genomic positions (e.g., −3 kb) from transcription start site (TSS, +1) are presented.

Regulatory sequences (extended promoter up to intron 1) of the 19 genes that showed transcriptional upregulation of corresponding proteins during hyperosmotic stress were obtained by searching the NCBI genome database for *O. niloticus*. Geneious prime 2022.0.1 (Biomatters) was used to extract and visualize approximately 5 kb of each of these 19 regulatory sequences (Figure 2). The criteria by which the regulatory sequences of each gene were selected from the downloaded NCBI sequence database were as follows: 1. Trim up to 5 kb long upstream regulatory region relative to transcription start site (TSS); 2. If the upstream regulatory region is overlapped with another gene body nearby, trim up to that point of the overlap; 3. The 5’ untranslated regions (UTRs) were included (such as exon 1 and intron 1). The last criterion we adopted in this study was to rationalize according to our previous publications elucidating where CREs (in particular, osmotically responsive CREs) are located. We previously identified seven osmotically responsive CREs (OSRE1) that were localized between −232 and +56 relative to the TSS and intron 1 in several osmotically regulated genes [17,18]. Thus, emphasis was placed on including this region for each of the 19 genes. 

**Figure 3:**
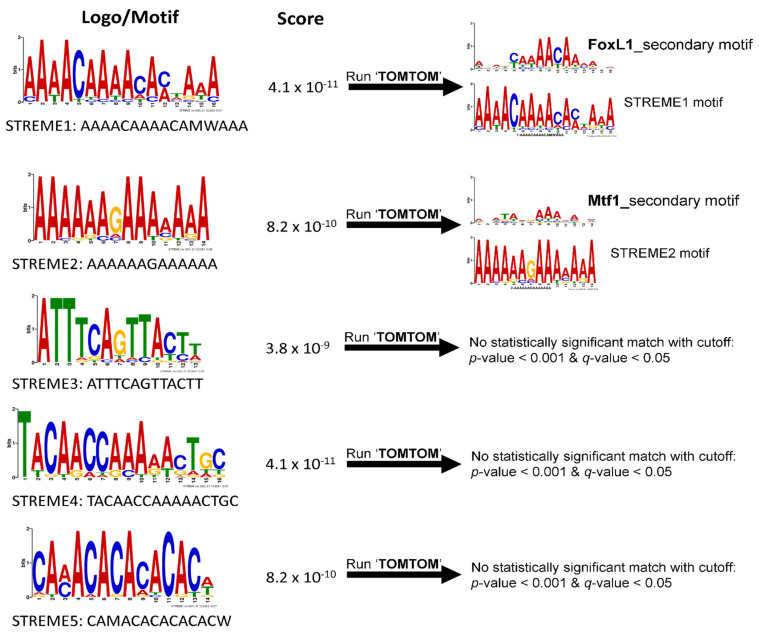
Five motifs (STREME1 to STREME5) identified by STREME motif discovery tool using 19 hyperosmotically induced tilapia sequences as input. The logo and sequence for each motif is indicated on the left and corresponding STREME score in the center. The result of TOMTOM prediction of known TF binding sites is indicated on the right. On the right are STREME1 motif results indicating a match to the FoxL1 secondary binding motif and STREME2 motif results indicating a match to the Mtf1 secondary motif from TOMTOM search. The other three STREME motifs did not match to any known TF binding motif with the cutoff criteria.

STREME analysis was performed on the 19 regulatory sequences to find putative hyperosmolality-responsive motifs that are enriched in the set of the regulatory sequences (Figure 3). STREME analysis yielded five motifs. For each of the resulting STREME motifs (STREME1 to STREME5), detailed information (e.g., logo, motif sequence, score) is shown in Figure 3. These five discovered motifs were then subjected to TOMTOM motif comparison analysis to see if any motif discovered by STREME resembles a previously known TF binding site (Figure 3). TOMTOM compares motifs against publicly known TF binding motif databases (e.g., JASPAR) and ranks the motifs in the database to produce an alignment for each significant match. This analysis revealed that STREME1 and STREME2 best match to the Forkhead box protein L1 secondary motif (FoxL1_2nd) and metal response element binding transcription factor 1 secondary motif (Mtf1_2nd), respectively. The other three motifs (STREME3, STREME4, and STREME5), however, yielded no statistically significant match with the cutoff values of *p*-value < 0.001 and q-value < 0.05 (Figure 3), indicating that these motifs are perhaps distinct in tilapia compared to the organisms included in the databases used by TOMTOM. Nevertheless, the TOMTOM-driven refinement process allowed prediction of putative transcription factor for two of the five discovered motifs (STREME1 and STREME2). 

### 3.3. Annotating STREM1 Hit Localization on the 19 Regulatory Sequences and Selecting for Candidate Gene Regulatory Regions to Be Experimentally Tested 

**Figure 4:**
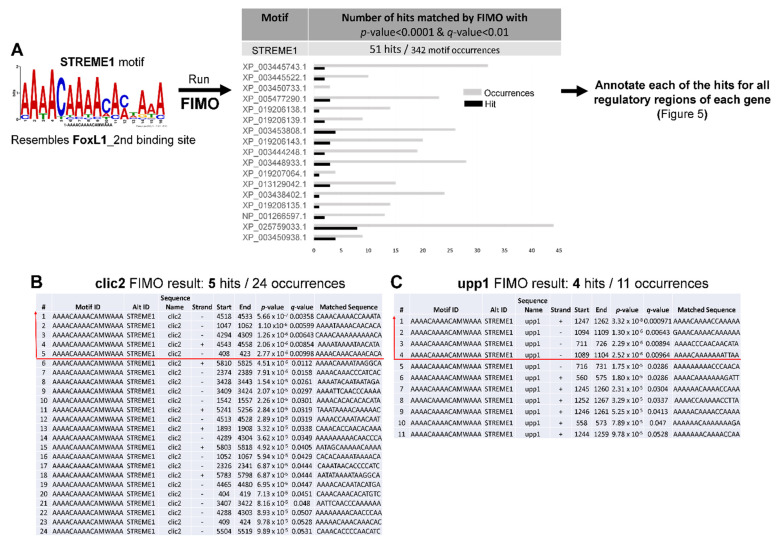
STREME1 motif scanning for occurrences in the regulatory sequences of 19 hyperosmotically induced tilapia genes. (**A**) FIMO was performed using the STREME1 motif to find all occurrences in the regulatory sequences. The sequence information for all identified STREME1 motifs in the regulatory sequences resulting from FIMO, such as whether STREME1 is found in sense or antisense strand; start and end position; *p*-value, and q-value are provided in supplementary Table S2. Fifty-one significant hits out of 342 total STREME1 occurrences were identified throughout all 19 regulatory sequences and were screened by two statistical cut off values using FIMO default *p*-value (0.0001) and q-value (0.01) thresholds. The number of occurrences of significant hits (black bars) and total STREME1 occurrences (grey bars) is illustrated for each of gene except for *clic2* and *upp1*, which were chosen for further experimental validation. Some occurrences of these 51 hits represent overlapping sequences, which were consolidated into a single motif in Figure 5. Detailed information about STREME1 motifs identified in *clic2* (**B**) and *upp1* (**C**) regulatory sequences is shown in panels B and C with significance thresholds indicated by a red line.

We chose to focus on the most highly significant motif, STREME1, for further analyses based on the results generated by STREME and TOMTOM. Next, we investigated STREME1 by performing FIMO analysis to scan all 19 regulatory sequences for occurrences of the STREME1 motif. This analysis revealed multiple occurrences in each sequence in total (342), 51 of which were statistically significant at *p*-value < 0.0001 and q-value < 0.01 (Figure 4). A complete list of the location of all motifs in each sequence is provided in Supplementary Materials (Table S2). 

**Figure 5:**
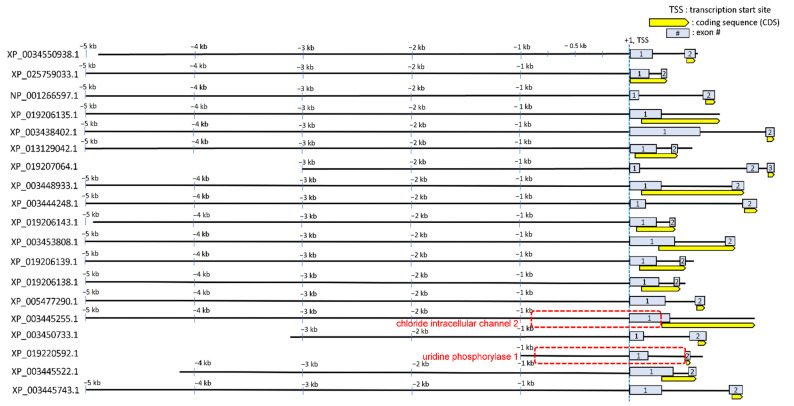
Annotation of significant STREME1 motif occurrences in regulatory sequences of 19 hyperosmotically induced tilapia genes. Each of the regulatory sequences analyzed by motif discovery is depicted as a black line. Light grey boxes indicate exons and the yellow boxes with one-sided arrow indicate coding sequence (CDS) including intron 1 if applicable. Significant hit STREME1 motifs (analyzed in Figure 4) are displayed using black bars with one-sided arrow indicating orientation. The proximal extended promoter sequences (approximately 1 kb upstream relative to TSS and 5’-UTR) for *clic2* and *upp1* that were used for experimental validation of STREME1 motifs are boxed by a dashed red line.

Due to the highest probability of STREME1 being a functional motif predicted by motif screening, significant occurrences of this motif detected by FIMO were annotated on each of the 19 regulatory sequences to visualize their genomic localization using Geneious Prime software (Figure 5). Then, we examined the regions including 1 kb upstream relative to TSS and 5’ UTR regions (including exon 1 and intron 1) to determine any enrichment pattern of the STREME1 motif in this region. The rationale for first focusing on this region was that proximal promoters, noncoding exon 1, and intron 1 were previously shown to harbor osmoresponsive CREs, which facilitate transcriptional induction during hyperosmolality [17,18]. Chloride intracellular channel 2 (*clic2*) and uridine phosphorylase 1 (*upp1*) each had three significant occurrences of the STREME1 motif in this region and were selected for experimental validation of the functionality of this motif during hyperosmotic stress. Since we used the genomic sequence of *O. niloticus,* but OmB cells were derived from *O. mossambicus*, these sequences were cloned and re-sequenced from *O. mossambicus* genomic DNA. As expected (the two species are very similar, forming fully functional and fertile hybrids in nature [48,49]), the pairwise identity between *O. mossambicus* and *O. niloticus* sequences for these regulatory sequences was very high—95.4% of 1037 bp for *clic2* and 96.2% of 1216 bp for *upp1*, respectively—and all STREME1 motifs were conserved (Figure S2).

### 3.4. Experimental Validation of the Selected Candidate Gene STREME1 Motifs 

**Figure 6:**
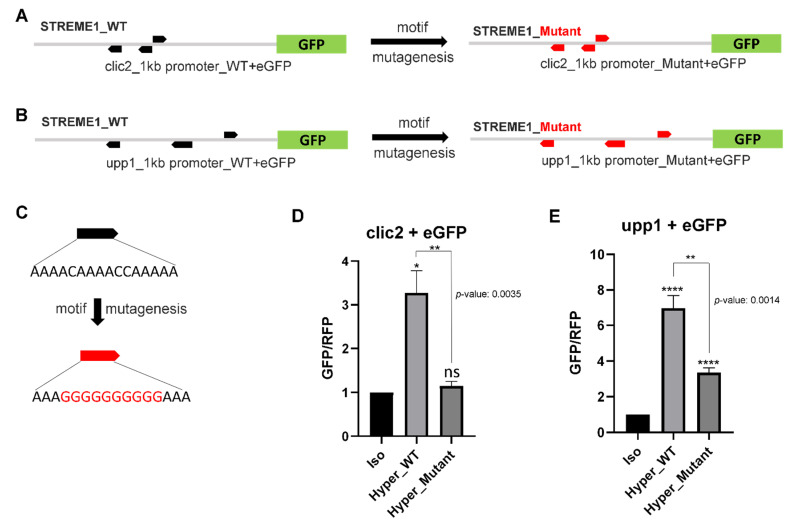
Experimental validation of STREME1 using GFP/RFP reporter assay and motif mutagenesis. Motif mutagenesis was used to replace the original STREME1 (black arrows) with a nonfunctional sequence (red arrows) by changing the core nucleotides with nucleotides not contained in that core region for chloride intracellular channel 2 (*clic2*, **A**) and uridine phosphorylase (*upp1*, **B**). (**C**) STREME1 motif mutagenesis strategy indicates the sequence difference between wild type (WT, black arrow) and mutant (red arrow) motifs. The transcriptional activities of the proximal regulatory regions from *clic2* (**D**) and *upp1* (**E**) during hyperosmolality (compared to isosmotic control medium) were measured by GFP signal (normalized with RFP control) using expression vector systems. t-test was used to calculate statistical significance yielding *p*-values. Iso: Isosmotic control, Hyper_WT: wild type regulatory sequence treated with hyperosmotic treatment, Hyper_Mutant: mutant regulatory sequence treated with hyperosmotic treatment, n = 5 (*: *p* < 0.05, **: *p* < 0.01, ****: *p* < 0.0001).

The proximal extended promoter sequences of two candidate genes, *clic2* and *upp1*, were PCR amplified and cloned into EGFP-reporter vector (Figure S3) to test their transcriptional activity during hyperosmolality. The comparative transcriptional activities were measured by GFP signals and tile scan images (RFP signal was used to normalize GFP signal) using the fluorescence microscope and subsequently quantified using the processing software installed. The approximate 1 kb proximal extended promoter sequences isolated from *clic2* and *upp1*, were shown to drive transcriptional induction in response to hyperosmolality (Figure 6A—left panel, Figure 6B—left panel, Figure 6D,E). 

To determine whether STREME1 is responsible for hyperosmotic inducibility of osmoregulated tilapia genes and to what extent it contributes to their regulation in response to hyperosmolality, the STREME1 wildtype and STREME1 mutant forms of each proximal extended promoter region (boxes surrounded by red lines in Figure 5) were analyzed using GFP/RFP reporter assay. Hyperosmotic induction of both *clic2* and *upp1* was confirmed using 1kb of their wildtype proximal extended promoter regions. Moreover, when all three STREME motifs were mutated to render them nonfunctional, the hyperosmotic inducibility of both genes was significantly reduced (Figure 6). Interestingly, the reduction was about two- to three-fold in both cases. However, since *upp1* hyperosmotic induction was much greater than *clic2* hyperosmotic induction, only mutation of *clic2* STREME1 motifs completely abolished hyperosmotic inducibility of the reporter. In contrast, reporter activity was still significantly higher in hyperosmotic medium for the upp1 mutant construct suggesting that this 1 kb regulatory sequence contains other osmotically responsive CREs in addition to STREME1. Overall, however, these results represent experimental validation of STREME1 as a novel salinity-responsive CRE in euryhaline tilapia. 

## 4. Discussion

### 4.1. The Role of CREs in Environmental Acclimation of Fish

In the present study, a sequential approach consisting of a multi-step bioinformatics methodology followed by experimental validation of the function of candidate sequences was used to identify a novel CRE (STREME1). Moreover, we predict the corresponding TF required for transcriptional activation of salinity-induced genes via STREME1 in euryhaline tilapia. We hypothesized that transcriptionally coregulated genes encoding hyperosmotically induced proteins have common regulatory elements that control their expression during hyperosmolality. Hyperosmolality-induced proteins in tilapia OmB cells were identified by quantitative proteomics and their transcriptional activation was verified using actinomycin D treatment. Transcriptional regulation in response to environmental cues such as hyperosmotic stress is largely governed by CREs and TFs [50,51]. For example, stress response CREs and stress-induced TFs that respond to a variety of stressors, such as heat shock, oxidative stress, and osmotic stress, have been dissected using the yeast model *Saccharomyces cerevisiae* [52]. Many studies have identified environmentally regulated genes, transcripts, and proteins, but many fewer have focused on the mechanisms by which CREs and TFs regulate mRNA and protein abundances. Nonetheless, some studies have attempted to elucidate environmentally induced transcriptional regulation of fish, including two studies from our lab that have identified osmolality/salinity responsive element 1 (OSRE1) as a CRE necessary for hyperosmotic induction of tilapia inositol monophosphatase 1 (IMPA1), *myo*-inositol phosphate synthase (MIPS), and glutamate synthetase (GS) genes [17,18]. In addition to previous studies, some CREs have been identified in other fish species. *PelB* enhancer (CRE) was identified as a major driver of *Pitx1* gene expression in the developing hind limb in sticklebacks. *Pitx1* encodes a homeodomain TF that controls hind limb development of the fish [53]. In zebrafish, a number of p63 TF binding sites (CREs) are located upstream of epidermal genes (e.g., *dlx3b*, *grhl1*, and *myh9a*) that are regulated as a p63-TF-controlled gene regulatory network [54]. Osmotic stress transcription factor 1 (OSTF1/TSC22D3) and TFIIB as salinity-induced TFs in tilapia whose induction precedes that of osmoregulatory effector genes were previously identified [55,56,57]. OSTF1 has since been confirmed as a rapidly osmoregulated gene in several other species of euryhaline fish [58]. In medaka intestine, OSTF1 mRNA is upregulated along with serum/glucocorticoid regulated kinase (SGK1) [59]. The importance of cis-regulatory elements for adaptive divergence of marine vs. freshwater sticklebacks was emphasized without specifying the specific cis-elements that are involved [60]. Our identification of a functional CRE (STREME1) and its putative transacting factorFoxL1 provides a new specific target for dissecting mechanisms of osmosensory signal transduction in euryhaline fishes. 

### 4.2. Transcriptional Regulation of Genes That Penetrates to Proteins and Phenotypes

In the present study, we have focused on hyperosmotically regulated proteins to emphasize corresponding genes whose regulation penetrates to phenotypes and take into account frequently observed lack of correlation of inducible mRNA versus protein abundance regulation [36,61,62,63,64]. This approach contrasts with many studies on fish that have been performed at the transcriptome level in response to different environmental signals, including changes in osmolality. One study examined the transcriptome profile of gill tissue of euryhaline estuarine goby, *Gillichthys mirabilis*, exposed to osmotic stress to identify osmotically responsive mRNAs. This study revealed many effector genes that encode putative osmosensory signaling proteins, including insulin receptor substrate-2 (IRS-2) and insulin-like growth factor binding protein 1 (IGFBP-1) [65]. Another study investigating the liver of spotted sea bass, *Lateolabrax maculatus,* challenged with salinity stress found 455 differentially expressed genes (DEGs) by RNA-seq, including many involved in cell signaling [66]. Deep sequencing of the gill transcriptome of hybrid tilapia exposed to salinity stress revealed many DEGs with signaling functions, e.g., carbonic anhydrase (CA), aquapoin-1 (AQP-1), and calcium/calmodulin-dependent protein kinase (CaM kinase) II [67]. However, these and many other transcriptomics studies do not identify the mechanism of mRNA abundance regulation, i.e., whether it is transcriptional or posttranscriptional, and they do not demonstrate that mRNA regulation penetrates to the level of proteins to affect phenotype. Our study demonstrates both by analyzing osmotic effects on protein abundance and by utilizing the specific transcription inhibitor actinomycin D [29]. This inhibitor has been used extensively for confirming transcriptional regulation of mRNA abundances, including in fish exposed to environmental stress. For instance, we previously utilized actinomycin D to investigate the mechanism of mRNA induction for OSTF1 in gills of tilapia exposed to hyperosmotic stress [55]. Another study used actinomycin D to show that hyperosmotic OSTF1 induction in gill cells of Japanese eels (*Anguilla japonica*) is in part due to transcriptional regulation [68]. 

Because phenotypes of cell lines can change with passage number, we have consistently used a narrow range of passages (20 to 27) of the OmB cell line. Nevertheless, we have previously documented that hyperosmotic stress response phenotypes of the OmB and OmL cell lines do not differ in their response to hyperosmolality and corresponding phenotype when low (passage 10 and 11) and high (passage 63) passages were compared [69]. 

### 4.3. STREME1 as a Novel Hyperosmotically Inducible CRE of Euryhaline Tilapia

Whether the predicted STREME1 motif is necessary for transcriptional regulation of *clic2* and *upp1* genes during hyperosmotic stress was experimentally tested. For both genes, mutagenesis of STREME1 significantly reduced the hyperosmotic inducibility. Intriguingly, STREME1 motif mutagenesis almost completely abolished hyperosmotic inducibility of the 1 kb proximal extended promoter region of *clic2* while that of *upp1* was only partially abolished after mutagenesis despite both regulatory regions being approximately equal in length and containing 3 STREME1 sites each. These data indicate that other CRE/TF binding sites are involved in hyperosmotic induction of *upp1*. For *clic2*, however, STREME1 plays a dominant role for the hyperosmotic activation. Combinatorial transcriptional regulation of *upp1* during hyperosmolality by multiple TFs is consistent with combinatorial regulation of many other genes in a diverse array of contexts as demonstrated in fruit flies, yeast, and mammals [70,71]. This cooperativity of multiple TFs with corresponding binding sites (CREs) has gained much attention as it can explain highly complex spatiotemporal transcriptional regulation [71]. Although combinatorial transcriptional regulation has been mostly studied in model organisms, there are some reports of combinatorial functions of TFs in fish. A study on the molecular mechanism of arterial formation investigating arterial-specific gene regulation in zebrafish has demonstrated that arterial specification is regulated by combinatorial binding of both the Notch and SOXF TFs [72]. Another zebrafish study on the involvement of ETS family TFs in early endothelial specification and differentiation elucidated that four members of this TF family (*fli1, fli1b, ets1, and etsrp*) function in combination with each other to achieve full vascular development, which was confirmed by introducing defective mutants of each gene [73]. 

Based on previous studies and those of others investigating CREs in several osmoresponsive genes in fish and mammals, respectively, a majority of CREs are localized within proximal promoter regions (within 1 kb upstream relative to TSS) or even intron 1 (5’ UTR) [17,18,74,75]. Consequently, we have focused on the approximately 1 kb extended promoter regions of *clic2* and *upp1* for experimental validation. However, other CREs contributing to the overall transcriptional regulation of hyperosmotically inducible genes are likely also involved in a combinatorial manner. For example, in mammals, salinity-responsive enhancers are scattered over a 50 kb region relative to the TSS [19]. Long-range inducible CREs have also been revealed for other contexts in diverse model species [10,21,76]. Thus, although the reporter studies utilized can unambiguously demonstrate that a particular CRE is necessary and contributes to the hyperosmotic regulation, it is not possible to conclude whether it is sufficient even if hyperosmotic induction is completely abolished by mutagenesis as is the case for the *clic2* 1 kb extended promoter.

### 4.4. Roles of CLIC2 and UPP1 during Hyperosmolality 

Sequences of chloride intracellular channel (CLIC) proteins are highly conserved among vertebrates but individual CLIC family members have multiple distinct cellular functions [77]. CLIC2 is the least studied CLIC family member. A mechanistic study of CLIC2 functions in human cancer tissues demonstrated that, apart from chloride transport, CLIC2 is involved in tight junction formation [78]. Tight junctions are known to be critical for osmoregulation, including in Mozambique tilapia gill epithelium [79]. Therefore, transcriptional regulation of CLIC2 upon hyperosmolality may be a physiological response that contributes not only to cellular osmoregulation but also to integrative osmoregulation at higher levels of organization [80]. 

The *upp1* gene encodes an enzyme that catalyzes the reversible phosphorolysis of deoxy-uridine and uridine to ribose phosphate and uracil, respectively [81]. The produced molecule is then utilized as a carbon and energy source or in the process of nucleotide synthesis [82]. Both uses can facilitate cellular osmoregulation and salinity stress responses of tilapia because substantial amounts of energy are required to cope with stressful conditions [83]. Moreover, it is necessary to produce more nucleotides including uracil (for *de novo* generating RNA molecules) to compensate for reduced nucleotide pools caused by stress-induced DNA and RNA damage [84,85]. The nonspecific nature of such effects of environmental stress on macromolecular damage and the induction of *upp1* during acute heat stress in black rockfish (*Sebastes schlegelii*) supports its role for replenishing building blocks for RNA pools during stress [86]. Moreover, the *upp2* gene of Javanese medaka (*Oryzias javanicus*), was shown to be induced by yet another type of stress, bisphenol A (BPA), which is a potent environmental toxicant, implicating *upp2* in the compensation of BPA chemical toxicant stress [87]. 

### 4.5. Other Candidate Binding Sites for Hyperosmolality Inducible TFs

Five other candidate motifs for hyperosmotically inducible CREs have been identified (STREME2-5) although, unlike STREME1, they have not been experimentally validated in this study. STREME2 was predicted to serve as a putative binding site for Mtf1. Known functional roles of Mtf1 include the activation of metal-induced expression of metallothionein (MT) genes [88,89]. Recently, it has been demonstrated that Mtf1 is involved in stress signaling and iron homeostasis in zebrafish [88], which supports our finding of Mtf1 as a potential TF involved in hyperosmotic stress responses. The other candidate motifs (STREME3, STREME4, and STREME5) identified did not meet the statistical significance threshold for any known TF. It is possible that these motifs are sufficiently different in fish from mammals and other organisms for which comprehensive TF binding motif databases are available. A common binding site shared by multiple STREME motifs is that for Sox TFs although none of the corresponding matches meets the significance threshold (Table S3). Sox TFs control development, cell survival, and physiological homeostasis [90]. 

TFs regulate the expression of genes having roles in a variety of environmental contexts through sequence-specific interactions with DNA and their DNA recognition specificity has been regarded as a crucial factor of transcriptional regulatory networks [91,92]. TF binding site databases document the binding preferences of TFs based on curated data from model organisms. Binding preferences of TFs from select model organisms to specific sequences have been extensively examined using protein binding microarray (PBM) technology, which assesses in vitro DNA binding preferences of TFs from yeast, mice, and humans [93,94,95]. These studies have demonstrated that distinct modes of DNA binding exist for many TFs and different (primary and secondary) motifs can be bound with potentially distinct regulatory functions that depend on the cellular environment. Our results show that the STREME1 motif matches the FoxL1 secondary binding site while it differs from the FoxL1 primary binding site, which is shared with other Fox TFs (GTAAACA). It has been suggested that the secondary binding specificity of FoxL1 has been acquired to permit usage of this TF in multiple contexts for controlling a variety of cellular processes throughout the evolution of transcriptional regulatory networks [96]. Thus, the secondary FoxL1 binding motif may have been favored during the evolution of transcriptional regulatory networks that control hyperosmotic stress responses [97].

### 4.6. Fox L1 as a Putative Hyperosmotically Inducible TF Binding to STREME1

Intraspecific comparative genomics approach has allowed for identification of STREME1 and its putative TF, FoxL1, as a CRE/TF duo necessary for the hyperosmotic induction of tilapia genes. The STREME1 motif (AAAACAAAACAMWAAA) contains the core sequence (CAAAACAA) of FoxL1 binding sites in mammals. In mammals, Fox family TFs, including FoxL1, have been described as important regulators of carcinogenesis [98] and stem cell differentiation [99]. Studying the effect of FoxL1 on the Wnt/β-catenin signaling pathway, Perreault et al. established that FoxL1 inhibits this pathway to deplete β-catenin in the nucleus, which in turn decreases cell proliferation in a *FoxL1*-null mouse model [100]. In contrast, another group demonstrated that FoxL1 can activate the same pathway by promoting the induction of tumor necrosis factor (TNF) related apoptosis-inducing ligand (TRAIL) in cancer cells [101]. Thus, in a mammalian system, FoxL1 TF has been shown to act as either activator or repressor depending on the specific combinatorial context, presumably defined by which other sets of TFs it interacts with. Interestingly, the Wnt/β-catenin pathway has been implicated in osmoregulation in tilapia [36]. In zebrafish, one study suggests that FoxL1 acts as transcriptional repressor of the sonic hedgehog (*shh*) gene, regulating central nervous system development [102]. This finding contrasts to our proposed role of FoxL1 as a transcriptional activator. However, little is known about physiological roles of FoxL1 in environmental stress responses and nothing about its function in the hyperosmotic stress response in fish. Moreover, as outlined above, depending on context, FoxL1 can also act as transcriptional activator. Furthermore, it is possible that in fish, other Fox family TFs bind to STREME 1 even though the STREME1 motif is most similar to the mammalian FoxL1 binding site. The TOMTOM-generated TF candidates identified in our study (Table S3) included not only FoxL1 for the STREME1 and STREME2 motifs, but also FoxK1 for the STREME2 and STREME5 motifs. FoxL1 and FoxK1 binding sites are highly similar, which renders both of these TFs strong candidates for hyperosmotically activated TFs in euryhaline fish. 

## 5. Conclusions

Using a bioinformatics approach based on intraspecific comparative genomics, we identified a novel hyperosmotically inducible CRE of euryhaline tilapia, STREME1. STREME1 function during hyperosmotic stress was experimentally validated using reporter assays in combination with site-directed mutagenesis of two different genes (*clic2* and *upp1*). Furthermore, FoxL1 and potentially its close ortholog FoxK1 were identified as candidate TFs that bind to STREME1 and possibly additional CREs (STREME2 and STREME5) in hyperosmotically regulated tilapia genes. This systematic approach consisting of intraspecific comparative genomics and experimental validation represents a powerful complement to widespread RNA-seq studies to identify the mechanisms by which stress-induced genes are regulated during specific environmental contexts. 

## Data Availability

All DIA proteomics data and metadata have been deposited and are publicly accessible at PanoramaPublic (accession https://panoramaweb.org/chk02.url, accession created on 9 March 2022) and ProteomeXchange (accession PXD032181) repositories.

## Supplementary Materials

The following supporting information can be downloaded at: https://www.mdpi.com/article/10.3390/life12060787/s1.
