# Supplementary material for: Prediction and Experimental Validation of a New Salinity-Responsive Cis-Regulatory Element (CRE) in a Tilapia Cell Line
Source: Life (Basel). 2022 May 25;12(6):787. doi: 10.3390/life12060787 (PMC9225295; doi:10.3390/life12060787)
Supplement: Supplementary file 1 [file life-12-00787-s001.zip › life-1712406-supplementary.pdf]

Supplementary Material

Title: Prediction and experimental validation of a new salinity-responsive *cis*-regulatory element (CRE) in tilapia cell line

Chanhee Kim[1], Xiaodan Wang[2] and Dietmar Kültz[1,*]

[1]Stress-induced Evolution Laboratory, Department of Animal Sciences, University of California, Davis, CA, 95616; cshkim@ucdavis.edu, dkueltz@ucdavis.edu
[2]Laboratory of Aquaculture Nutrition and Environmental Health, School of Life Sciences, East China Normal University, Shanghai, 200241, PR China; xdwang@bio.ecnu.edu.cn
*Correspondence: dkueltz@ucdavis.edu; Tel.: +1 530 752 2991

Supplementary Figures:

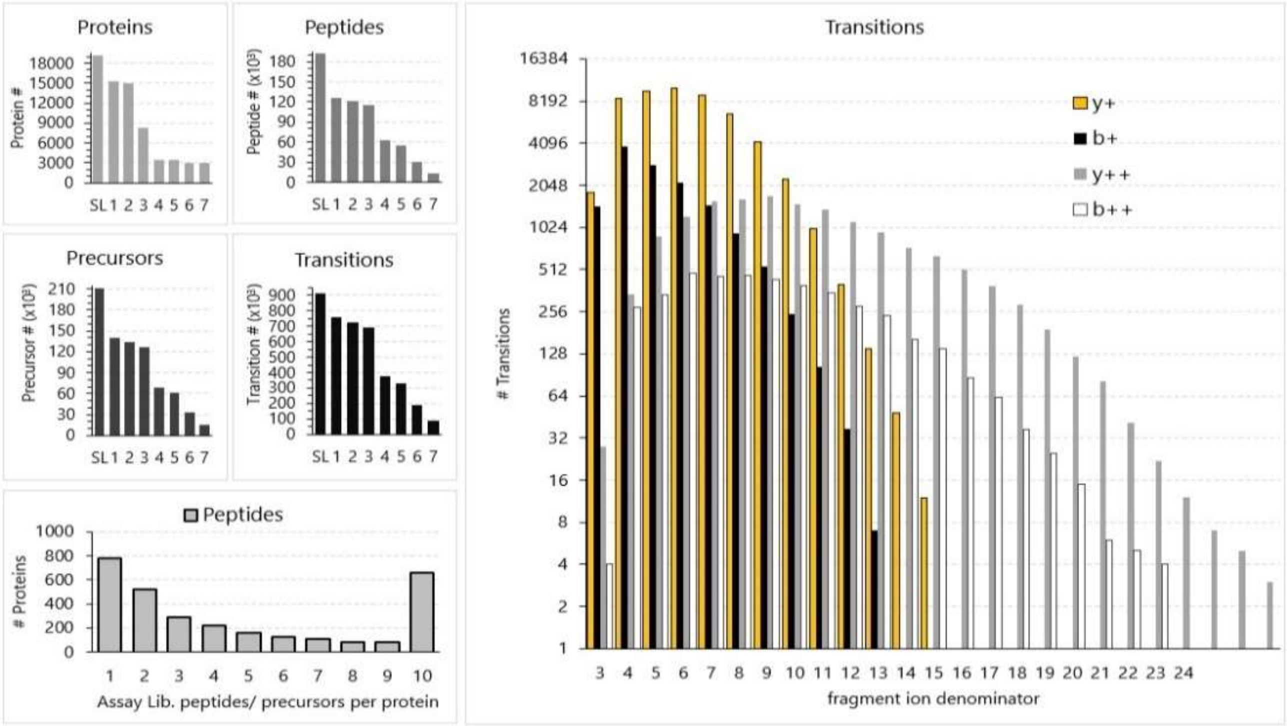

Figure S1: <u>DIA assay library of *Oreochromis mossambicus* OmB cells</u>. The initial spectral library (SL) contains over 18,000 proteins, 200,000 precursors, and almost 1 million transitions. Seven quality control (QC) filters were applied to create the DIA assay library containing 3043 unique proteins, 15,211 precursors (peptides), and 87,184 diagnostic transitions. Most proteins are represented by at least 2 diagnostic peptides. The remainder (25%) was identified by at least 2 unique peptides but only 1 remains after applying all DIA QC filters.

A. chloride intracellular channel 2 (clic2)_Wild Type

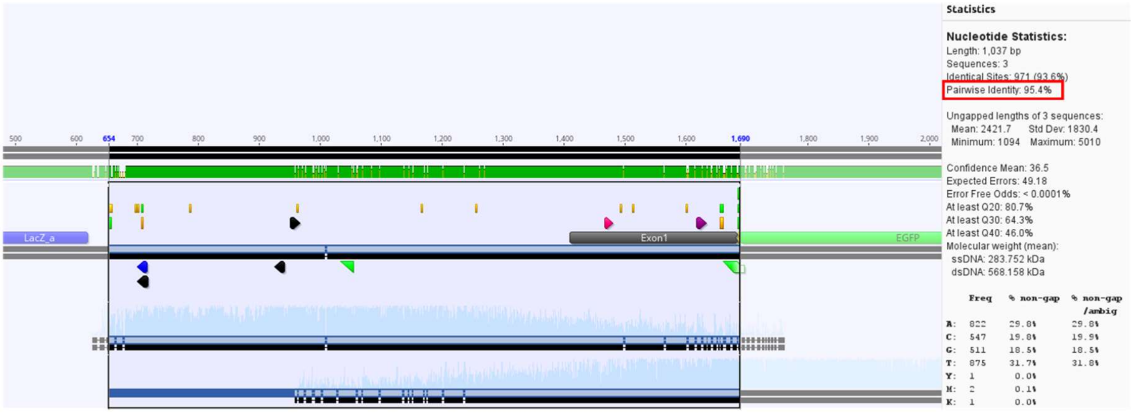

B. chloride intracellular channel 2 (clic2) _STREME1 motif mutant

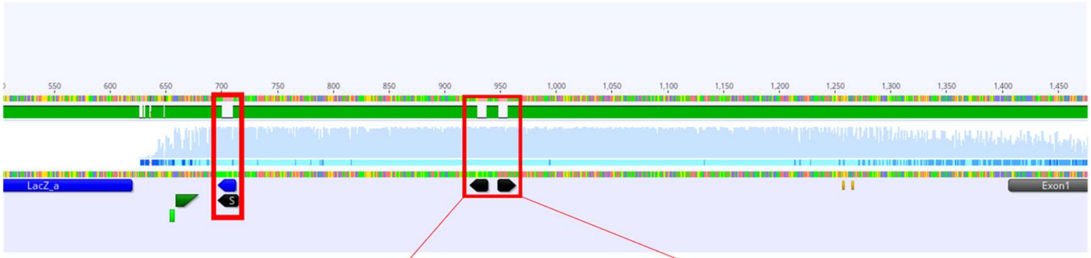

C. clic2_STREME1 motif mutagenesis representative result

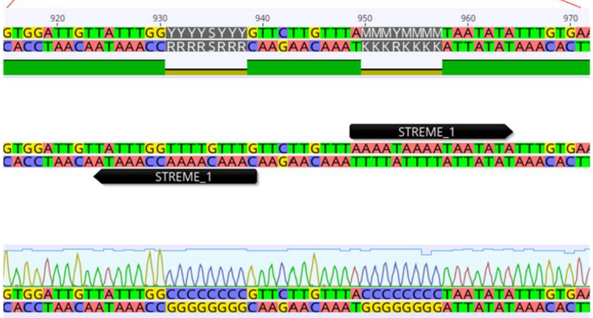

D. uridine phosphorylase 1 (upp1)_Wild Type

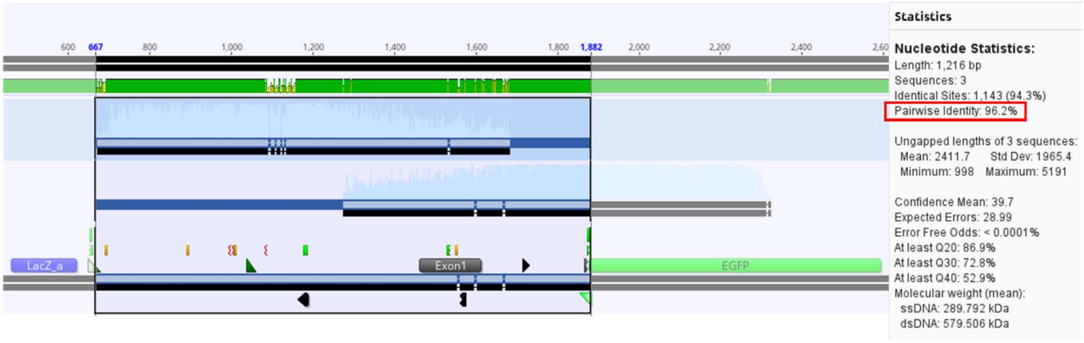

E. uridine phosphorylase 1 (upp1)_STREME1 motif mutant

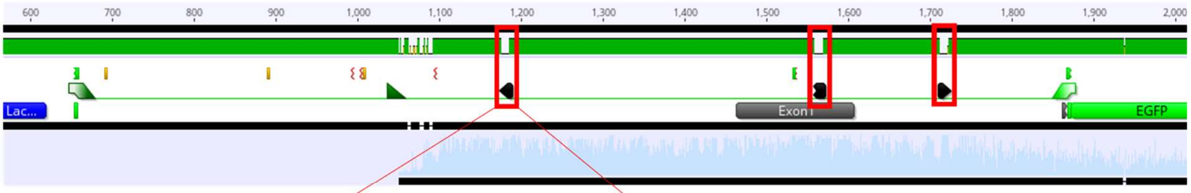

F. upp1_STREME1 motif mutagenesis representative result

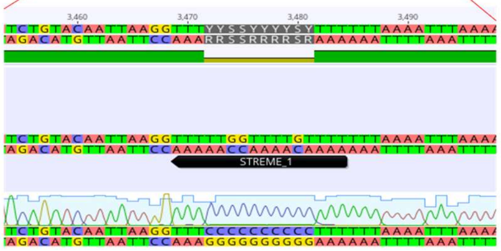

Figure S2: Sequence information of wild-type and mutant versions of approximately 1kb long proximal extended promoter regions of *clic2* and *upp1* that were used for quantitative GFP reporter assays. (A) The pairwise identity of *clic2* sequences between *Oreochromis mossambicus* and *Oreochromis niloticus* (reference genome) is 95.4 % as marked by red box. (B) STREME1 motifs found in the 1 kb promoter region of *clic2* were mutated using site-directed mutagenesis and confirmed by Sanger sequencing as indicated by red boxes. (C) A representative result of mutagenesis was shown with distinct chromatograms between wild-type reference (upper, STREME1 annotated) and mutant form (bottom). (D) The pairwise identity of *upp1* sequences between *Oreochromis mossambicus* and *Oreochromis niloticus* (reference genome) is 96.2 % as marked by red box. (B) STREME1 motifs found in the 1 kb promoter region of *upp1* were mutated using site-directed mutagenesis and confirmed by Sanger sequencing as indicated by red boxes. (C) A representative result of mutagenesis was shown with distinct chromatograms between wild-type reference (upper, STREME1 annotated) and mutant form (bottom).

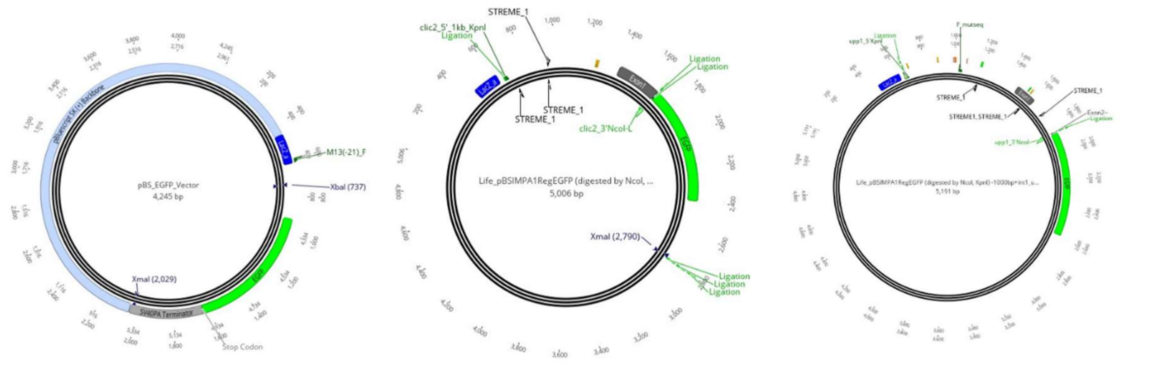

pBS_EGFP   pBS_EGFP +clic2_1kb (WT)   pBS_EGFP +upp1_1kb (WT)

Figure S3: <u>The schematic maps of the plasmids constructed for testing two candidate regions of *clic2* and *upp1*.</u> pBS_EGFP (basic backbone vector) is displayed at the left side, clic2_1kb_proximal extended promoter cloned pBS_EGFP (pBS_EGFP+clic2_1kb (WT)) at center, and upp1_1kb_proximal extended promoter cloned pBS_EGFP (pBS_EGFP+upp1_1kb (WT)) at the right side.

Supplementary Tables:

Table S1: Primer sequence information used in this study.

Primer Purpose	Primer Name	Primer Sequence
Wildtype PCR and Cloning	clic2_5'KpnI	CCCCCGGTACCCCTAATCAGAAGGATGCAGA
	clic2_3'NcoI	CCCCCCCATGGTTTATCCCCCAAAAGTTTTTGTTT
	upp1_5'KpnI	CCCCCGGTACCGTCCTGAACTGAAGTGATG
	upp1_3'NcoI	CCCCCCCATGGTTCCTGCGAACAGCCGAA
	clic2_5'KpnI	CCCCCGGTACCCCTAATCAGAAGGATGCAGA
Mutant PCR and Cloning	clic2_1p_3'overlapped	GCATTAGCAAAGGGGGGGGGGAAAAAACCAAGCTGAAGTTCATTA
	clic2_2p_5'overlapped	GCTTGGTTTTTTCCCCCCCCCCTTTGCTAATGCTATTGTTTTCGAA
	clic2_2p_3'overlapped	GCATTAGCAAAGGGGGGGGGGAAAAAACCAAGCTGAAGTTCATTA
	clic2_3p_5'overlapped	GCTTGGTTTTTTCCCCCCCCCCTTTGCTAATGCTATTGTTTTCGAA
	upp1_1p_3'overlapped	AGTGCCGGAATAAAAAAAAAGGGGGGGGGGATATAGCTGGC
	upp1_2p_5'overlapped	CCAAGGCCAGCTATATCCCCCCCCCCTTTTTTTTATTCCG
	upp1_2p_3'overlapped	CACGAAACCAAGAAGGGGGGGGGGAAAATTAAAAAGAAGATACAGAT
	upp1_3p_5'overlapped	TATCTTCTTTTTAATTTTCCCCCCCCCCTTCTTGGTTTCGTGAGTC
	upp1_3p_3'overlapped	AGATCTGTACAATTAAGGTTTCCCCCCCCCCTTTTTTAAAATTTAAAAC
	upp1_4p_5'overlapped	TTAAATTTTAAAAAAGGGGGGGGGGAAACCTTAATTGTACAGATCTGAT
Sequencing	M13_Forward	TGTAAAACGACGGCCAGT
	GFP_R5	TTCTTCTGCTTGTCGGCCAT

Table S2: STREME1_FIMO search result of the 19 regulatory regions for both occurrences and hits- see the attached Microsoft Excel Spreadsheet file.

Table S3: Lists of TFs for STREME motifs searched by TOMTOM- see the attached Microsoft Excel Spreadsheet file.
